# Supplementary figures and images for: Zeaxanthin dipalmitate-enriched wolfberry extract improves vision in a mouse model of photoreceptor degeneration
Source: PLoS One. 2024 May 20;19(5):e0302742. doi: 10.1371/journal.pone.0302742 (PMC11104671; doi:10.1371/journal.pone.0302742)

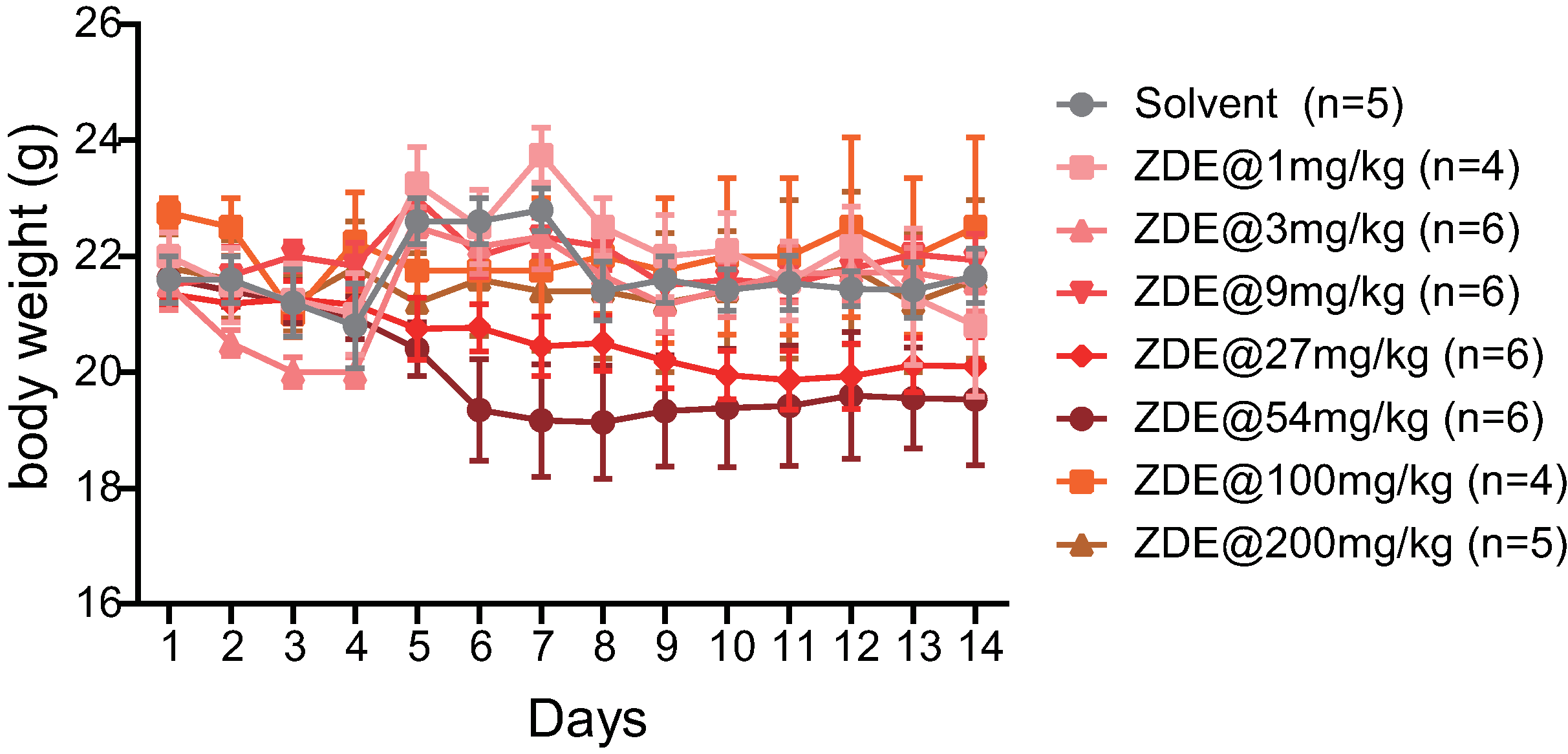

Supplement: S1 Fig — The record of mice’s body weight over 2 weeks of ZDE treatment at various doses. (TIF) [file pone.0302742.s001.tif]

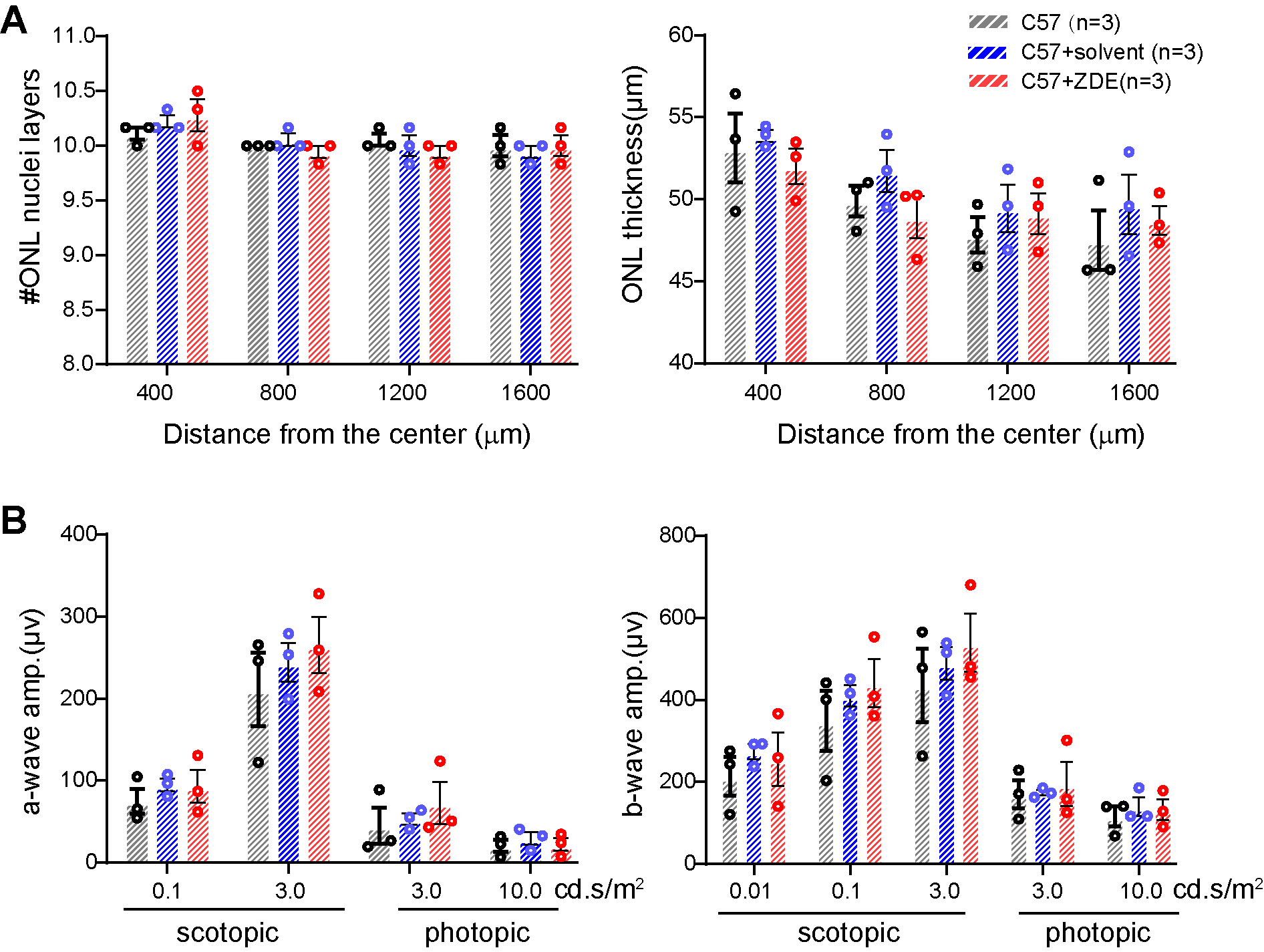

Supplement: S2 Fig — A. Average number of ONL nuclei layers (left) and thickness of ONL (right) of normal C57 retina and those treated with solvent or 9 mg/kg ZDE. B. Average amplitudes of a-wave (left) and b-wave (right) for normal C57 mice and those treated with solvent or 9mg/kg ZDE. (TIF) [file pone.0302742.s002.tif]
